# Enzyme‐responsive macrocyclic metal complexes for biomedical imaging

**DOI:** 10.1002/btm2.10478

**Published:** 2022-12-21

**Authors:** Quoc‐Viet Le, Jaiwoo Lee, Seungbeom Ko, Hyunjung Kim, Thien Y Vu, Yearn Seong Choe, Yu‐Kyoung Oh, Gayong Shim

**Affiliations:** ^1^ Faculty of Pharmacy Ton Duc Thang University Ho Chi Minh City Vietnam; ^2^ College of Pharmacy and Research Institute of Pharmaceutical Sciences Seoul National University Seoul Republic of Korea; ^3^ Department of Nuclear Medicine, Samsung Medical Center Sungkyunkwan University School of Medicine Seoul Republic of Korea; ^4^ Department of Health Sciences and Technology, SAIHST Sungkyunkwan University Seoul Republic of Korea; ^5^ School of Systems Biomedical Science and Integrative Institute of Basic Sciences Soongsil University Seoul Republic of Korea

**Keywords:** biomedical imaging, contrast agent, macrocyclam, metal ion

## Abstract

Metal chelator‐based contrast agents are used as tumor navigators for cancer diagnosis. Although approved metal chelators show excellent contrast performance in magnetic resonance imaging (MRI), large doses are required for cancer diagnoses due to rapid clearance and nonspecific accumulation throughout the body, which can compromise safety. The present study describes an enzyme‐responsive metal delivery system, in which enzyme overexpressed in the tumor microenvironment selectively activates the tumor uptake of gadolinium (Gd). Gd was loaded into enzyme‐responsive macrocyclam (ErMC) modified with a PEGylated enzyme‐cleavable peptide resulting in Gd@ErMC. The PEGylated shell layer protected Gd@ErMC from nonspecific binding in the blood, increasing the half‐life of the contrast agent. Specific cleavage of the PEGylated shell layer by the enzyme selectively liberated Gd from Gd@ErMC at the tumor site. Evaluation of the in vivo distribution of Gd@ErMC in tumor‐bearing mice by MRI and positron emission tomography (PET) showed that Gd@ErMC had an extended half‐life and was highly specific. Histological and serological analysis of Gd@ErMC‐treated mice showed that this agent was safe. This novel enzyme‐responsive contrast agent delivery system shows promise as specific theranostic agent for MR‐guided radiotherapy.

## 1 INTRODUCTION

Early diagnosis of cancer is important for effective treatment. Imaging and navigation of a tumor are required for more precise surgery, as well as for patient monitoring and managing response to treatment.^1^ Contrast agents based on metal chelators have been approved for the clinical detection of tumors.^2^ Gadolinium (Gd)‐based contrast agents (GBCAs) are currently used for early diagnosis of cancer because of their contrast performance in magnetic resonance imaging (MRI) and their acceptable safety profile.^3^ These GBCAs, however, have drawbacks, including nonspecific accumulation in normal tissue and rapid clearance due to their small molecular size. Large doses of Gd are therefore required for tumor detection, giving rise to safety concerns.^4^

The contrast performance of Gd has been enhanced by using nanoparticles.^5^ Nanoparticles not only increase the volume of the Gd payload but extend the blood circulation time of Gd. Various types of nanoparticles have been developed for MR imaging, such as gold‐based, iron oxide, and silica nanoparticles.^3^, ^6^, ^7^ Although these inorganic Gd delivery platforms can overcome the short half‐life and low contrast performance of Gd, concerns have arisen about their biocompatibility and the nonspecificity of signals.

Beside another major challenge of conventional GBCA is the nonspecific accumulation at normal organs as well as poor delivery to solid tumor. The systemic delivery of these GBCAs to tumor site is dominant by the passive diffusion mechanism, which result in broad distribution and low contrast performance. Their short half‐life leads to poor survival in blood and thus reducing the accumulation at tumor. In order to improve tumor‐targeting efficacy, it is required to overcome three issues: the low payload of contrast metal per molecule, low nonspecific uptake at normal tissue, and poor tumor specificity. Tumor microenvironment (TME)‐triggered activation may provide specific drug delivery to tumors. Fibroblast activation protein, matrix metalloproteinases (MMP), and hyaluronidase are enzymes more highly expressed in tumor tissue than in adjacent normal tissue.^8^, ^9^ The incorporation of enzyme‐cleavable peptides into nanoparticles can provide a method of specifically triggering drug release in the TME.^10^, ^11^ Modifications of contrast agents that enhance their TME‐homing features can therefore enhance their tumor specificity.

**FIGURE 1:**
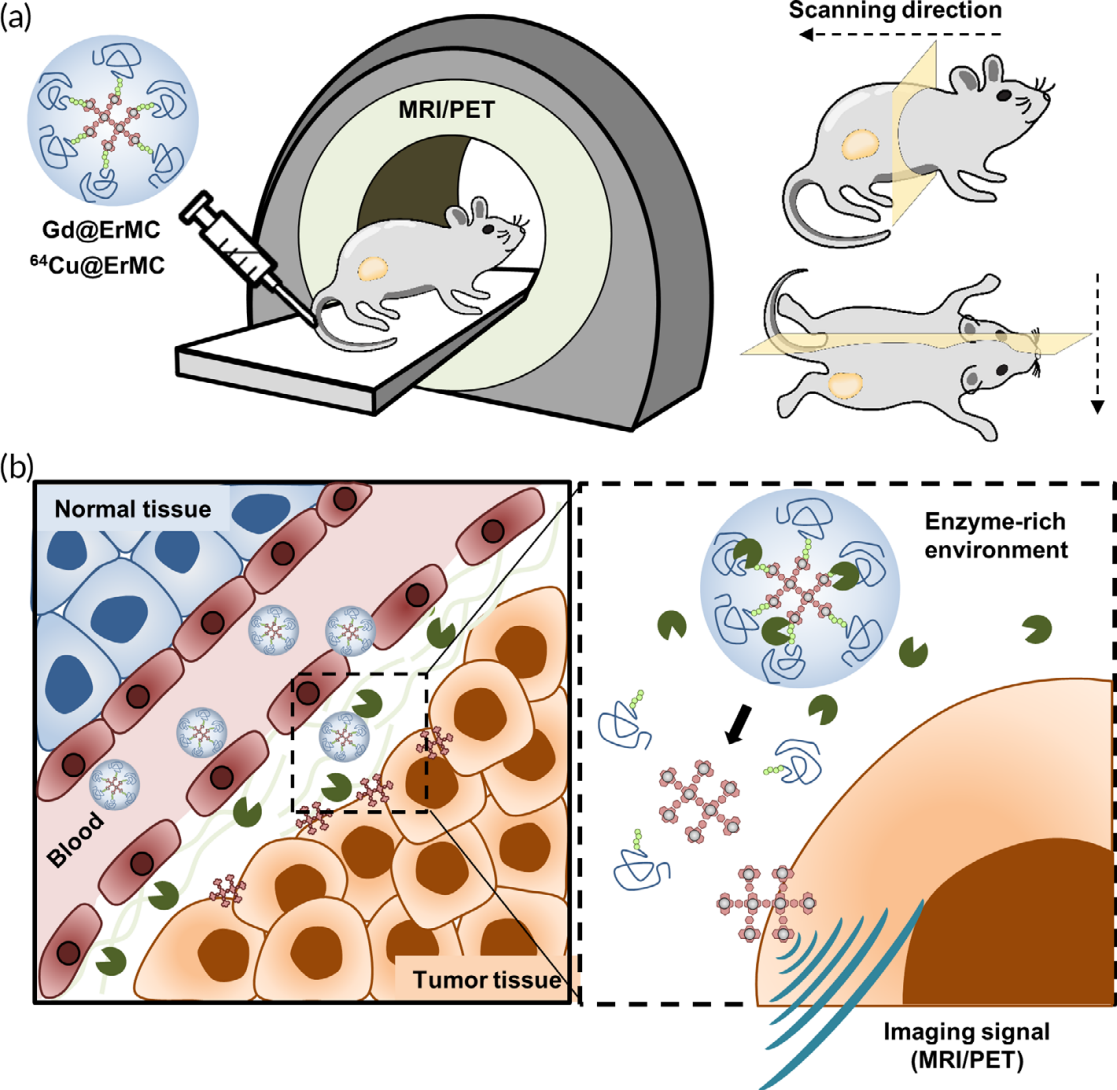
Proposed mechanism by which a macrocyclam‐metal complex acts as an enzyme responsive contrast agent. (a) Chelation of metal ions in macrocyclams masked with enzyme‐responsive PEG clouds, yielding enzyme‐responsive macrocyclams (ErMCs). Depending on the type of metal ion, ErMCs can be utilized as dual imaging probes for magnetic resonance (MR) and positron emission tomography (PET) imaging. (b) Activation of ErMC in enzyme‐enriched target tissues facilitating the specific accumulation of contrast agents.

The present study describes the synthesis of an enzyme‐responsive macrocyclam (ErMC) by modification with a PEGylated MMP‐cleavable peptide. Due to the hydrophobic nature of the cyclam structure, ErMC could self‐assemble into polymeric nanoparticles. Pegylation provided a protective shell to enhance the half‐life of ErMC in the blood circulation and prevent its nonspecific binding to blood cells and proteins. Once they are delivered to tumor tissue through passive penetration, the tumor‐matrix degrading enzyme trigger the deshielding of PEG shell layer via peptide cleavage and thus exposing the macrocyclam core/Gd complex. The naked complex is thus expected to improved tumor cell internalization and resident time at tumor tissue, which are important for an effective contrast performance. ErMC was loaded with gadolinium by coordination with the cyclam rings, resulting in the formation of Gd@ErMC. Systemic administration of Gd@ErMC into mice bearing enzyme‐rich tumors extended the half‐life of Gd in these mice and enhanced the performance of the MRI contrast agent (Figure 1).

## 2 MATERIALS AND METHODS

### 2.1 Synthesis of macrocyclam derivatives

Macrocyclam derivatives were synthesized using monocyclam (1,4,8,11‐tetraazacyclotetradecane, Sigma‐Aldrich, St. Louis, MO, USA) as a starting material.^12^ The synthesis scheme is illustrated in Figure S1. A solution of compound 1 in dichloromethane was added to Boc_2_O (1.8 equiv.) in dichloromethane and the mixture was stirred at room temperature for 6 h. The solvent was removed under reduced pressure, and the resulting mixture purified by silica gel column chromatography, with the column eluted with 10% EtOAc‐MeOH. A solution of compound **2** in acetonitrile was added to K_2_CO_3_ (2 equiv.) and 1,4‐bis(bromomethyl)benzene (1.2 equiv.), and the mixture was stirred at room temperature for 2 days. The reaction mixture was filtered off and the solvent was removed under reduced pressure. The resulting mixture was purified by silica gel column chromatography, with the column eluted with 40% hexane‐EtOAc. A solution of compound **3** in acetonitrile was added to K_2_CO_3_ (2 equiv.) and compound **2** (0.9 equiv.) and the mixture was stirred at room temperature for 2 days. The reaction mixture was filtered off, and solvent was removed under reduced pressure. The resulting mixture was purified by silica gel column chromatography, with the column eluted with 50% hexane‐EtOAc. Compound **4** was dissolved in dichloromethane and stirred with TFA at room temperature for 2 h. The solvent was removed under reduced pressure, and excess TFA was removed under high vacuum overnight at room temperature. A solution of compound **5** in acetonitrile was added to K_2_CO_3_ (12 equiv.) and compound **2** (8 equiv.) and the mixture was stirred at 60°C for 24 h. The reaction mixture was filtered off and the solvent was removed under reduced pressure. The reaction mixture was dialyzed against deionized water and methanol for 48 h. The solvent was removed under reduced pressure and high vacuum overnight at room temperature. Compound **6** was dissolved in dichloromethane and stirred with TFA at room temperature for 2 h. The solvent was removed under reduced pressure, and excess TFA was removed under high vacuum overnight at room temperature to produce compound **7**.

**FIGURE 2:**
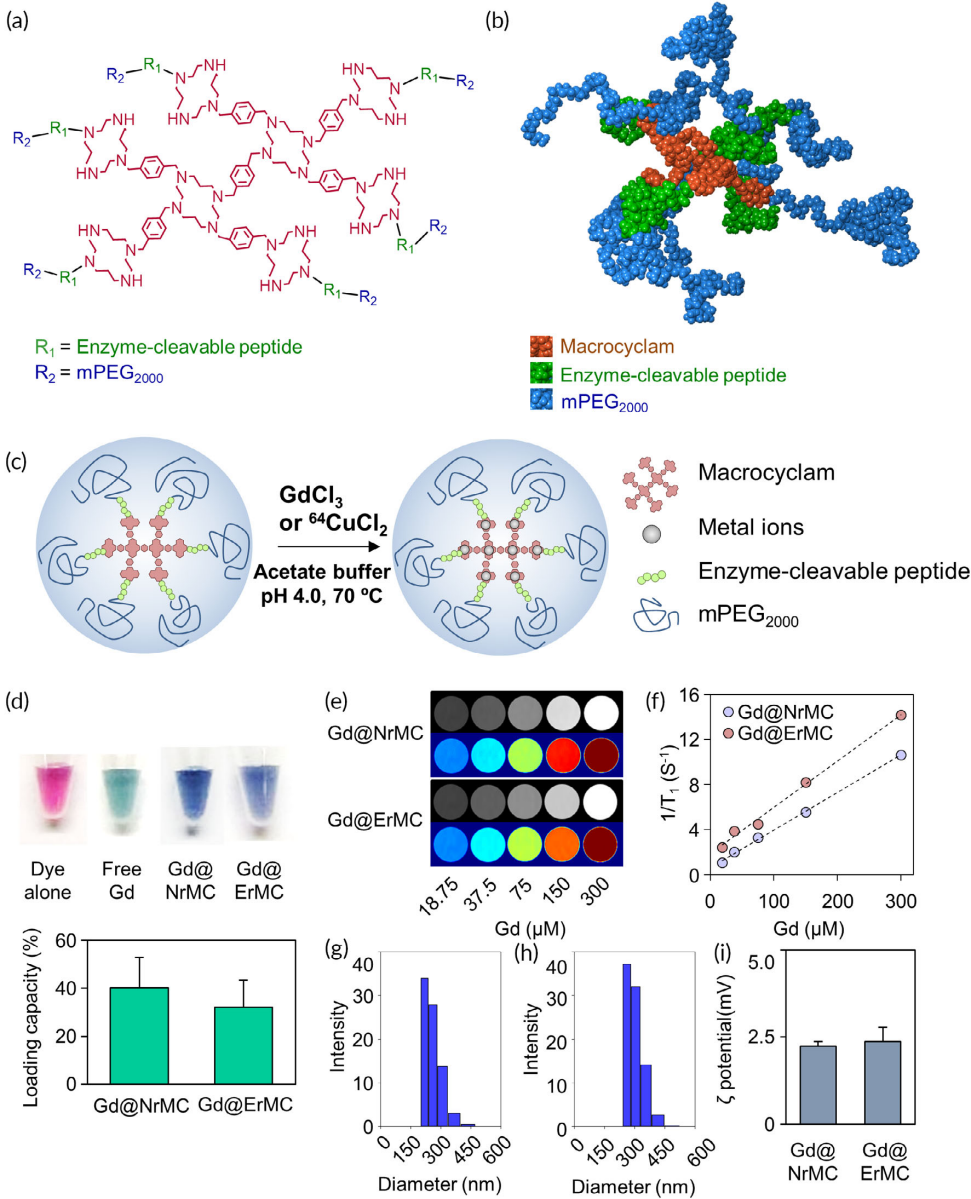
Physical and chemical characteristics of macrocyclam‐based contrast agent. (a) Chemical structure of enzyme‐responsive macrocyclam (ErMC). (b) Molecular dynamic simulation of ErMC. (c) Scheme of metal ion chelation with ErMC. (d) Determination Gd chelation in the complex by arsenazo III colorimetric assay and determination of Gd‐loading capacity by inductively coupled plasma mass spectrometry (ICP‐MS). (e) Concentration‐dependent T1‐weighted MR imaging of Gd in formulations. (f) Graph of Gd^3+^ concentration relative to the relaxation rate. (g, h) Size distributions of (g) Gd@NrMC and (h) Gd@ErMC. (i) Zeta potentials of Gd@NrMC and Gd@ErMC

The enzyme‐responsive peptide GGPLGVRGGGGG‐OH was synthesized by Peptron (Daejeon, Republic of Korea). Methoxypolyethylene glycol (mPEG) 2000‐NHS (NOF, White Plains, NY, USA) was conjugated to the N‐terminal of peptides by adding a 10% molar excess of mPEG‐NHS to peptide with diisopropylethylamine in DMF. After incubation for 24 h at room temperature, the products were cleaved with 2% TFA in DCM and the solvent was removed under reduced pressure and high vacuum overnight at room temperature. A solution of compound **6** in DMF was added to the above peptide conjugates (20 equiv.) and DIPEA (40equiv.) and the mixture was stirred at room temperature for 72 h. The solvent was removed under reduced pressure. The reaction mixture was dialyzed against deionized water and methanol for 48 h. The solvent was removed under reduced pressure and high vacuum overnight at room temperature. The resulting product (ErMC) was deprotected by mixing with 95:2.5:2.5 (vol/vol/vol) trifluoroacetic acid:H_2_O:triisopropylsilane, followed by precipitation with cold diethyl ether. The resulting product was washed several times with cold diethyl ether, dissolved in water, lyophilized, and stored as a lyophilized powder at −20°C. To produce enzyme nonresponsive macrocyclam (NrMC) (Figure 2a), compound **7** in DMF was added to mPEG2000‐NHS (20 equiv.) and DIPEA (40 equiv.) and the mixture was stirred at room temperature for 72 h. The solvent was removed under reduced pressure. The reaction mixture was dialyzed against deionized water and methanol for 48 h. The solvent was removed under reduced pressure and high vacuum overnight at room temperature.

### 2.2 Molecular dynamic simulation

The structure of the macrocyclam derivatives was constructed using Polymer Builder in Maestro Materials Science 3.9 of Schrodinger Suites (Figure 2b). The structures of the central cyclams were added manually, and the sequences of the peptides and PEG chains were combined and repeated automatically. A 10,000 molecule system with a polymer/water ratio set at 7/9993 was placed in the Disorder System, which was accepted for experimental concentrations and simulation system compatibility. The “tangled chain” option was selected and the force field was OPLS3e. The disordered system was subjected to a 100‐ns MD by using the multistage MD workflow of Desmond. Briefly, the *Compressive* relaxation protocol, which was shown effective for low‐density systems, was applied to the first MD stage. The next MD stage, which included a simulation time of 100 ns, a trajectory recording interval of 1000 ps, the NPT ensemble and a 2‐fs time step, was performed at a temperature of 300 K and a pressure of 1.01325 bar.

### 2.3 Complexes of metals with macrocyclam derivatives

Metal ions are complexed with macrocyclam derivatives in acidic condition (Figure 2c). The lyophilized macrocyclam derivatives were dissolved in distilled water. A 100 nmol aliquot of NrMC or ErMC was mixed with gadolinium chloride (Sigma‐Aldrich) or radioactive copper‐64 chloride in sodium acetate buffer (pH 5.0) and incubated for 2 h at 80°C. Unloaded Gd was removed using a PD‐SpinTrap™ G‐25 column (GE Healthcare, Little Chalfont, UK). The loading amount of Gd in each sample was measured by inductively coupled plasma mass spectrometry (ICP‐MS, Varian 820‐MS; Varian, Palo Alto, CA, USA). Gd chelation consists of a reaction between the Gd^3+^ and arsenazo III dye under acidic conditions to form a blue–purple complex. Radioactivity was measured using a dose calibrator (Biodex Medical Systems, Shirley, NY, USA).

### 2.4 Characterization

The size, zeta potential, and morphology of each macrocyclam derivative were determined. Size was measured using dynamic light scattering, and zeta potential was assessed by laser Doppler microelectrophoresis at an angle of 22°, with both size and zeta potential being measured using an ELS8000 instrument (Photal, Osaka, Japan). The relaxation times of Gd‐loaded materials were examined using a 7 T/20 MRI instrument (Bruker‐Biospin, Ettlingen, Germany) at 37°C. Each sample was serially diluted to Gd concentrations of 37, 75, 150, and 300 μM, and the T1‐weighted MR signal intensities of 200‐μl samples were determined. The relaxation rate, *r*1 (*r*1 = 1/T1), for each preparation was calculated from acquired images.

### 2.5 Cytotoxicity

The viability of cells treated with macrocyclam derivatives was assessed by MTT and apoptosis detection assays, and by live/dead cell staining. The 3T3‐L1 cells were seeded onto 24‐well plates at a density of 1 × 10^5^ cells per well for 24 h and the cells were treated with macrocyclam derivatives for 24 h. MTT (3‐(4,5‐dimethylthiazol‐2‐yl)‐2,5‐diphenyltetrazolium bromide) solution (final concentration, 500 μM) was added to each well and the cells incubated for 2 h. After aspiration of medium, the cells were dissolved in 200 μl dimethyl sulfoxide (DMSO; Sigma‐Aldrich). Formazan crystals generated by metabolically active cells were quantified by measuring optical density at 570 nm using a microplate reader (Tecan Group Ltd., Seestrasse, Mannedorf, Switzerland). Cell viability was analyzed by calculating the percent optical density of treated cells relative to control cells. Live and dead cell populations were determined using EZ‐view Live/Dead Cell Staining Kits (Biomax, Seoul, Republic of Korea). Briefly, calcein‐AM (final concentration 6.7 μM) and propidium iodide (PI) (final concentration 15 μM) were added to each well and incubated for 20 min at 37°C. The absorbance of each well was measured at 490/545 nm using fluorescence microscopy (IX70; Olympus, Japan). Cell apoptosis was analyzed using a FACSCalibur flow cytometer (BD Biosciences, CA, USA) with annexin V staining. Using the FITC Annexin V Apoptosis Detection Kit I (BD Pharmingen, Heidelberg, Germany), the cells were stained with 1 unit/well of two fluorescent dyes, PI and FITC Annexin V, for 15 min at 25°C in the dark. Cell surface expression was analyzed using a FACSCalibur flow cytometer (BD Biosciences), with the results analyzed using FlowJo software (BD Biosciences).

### 2.6 Cellular uptake

Cellular uptake was assessed by in vitro MR imaging, ICP‐MS, and scanning electron microscopy combined with energy‐dispersive x‐ray spectroscopy (SEM/EDS) analysis. For in vitro MR imaging, murine squamous cell carcinoma SCC7 cells were seeded onto six‐well plates at a density of 3 × 10^5^ cells per well. After 24 h, the cells were treated with Gadovist or macrocyclam derivatives at Gd concentrations of 100 μM for 24 h. MR images were obtained and analyzed using an MRI scanner (Bruker‐Biospin). To assess enzyme‐dependent cellular uptake, 3T3‐L1 cells were pretreated with various concentrations of MMP‐9 (Sigma‐Aldrich). For ICP‐MS analysis, the cells were washed and completely dissolved with 90% nitric acid (Sigma‐Aldrich). To quantify Gd taken up by cells, the dissolved cells were digested with 1 ml of aqua regia (HCl: HNO_3_ = 3: 1, m/m) at 130°C. The solutions were completely dried and redissolved in deionized water, and the amounts of Gd were measured by ICP‐MS (Varian 820‐MS). For SEM/EDS analysis, SCC7 cells were seeded in cover glass‐inserted 24‐well plates at a density of 2 × 10^5^ cells per well, followed by treatment of the samples for 24 h. A field‐emission SEM system (Supra 55VP; Carl Zeiss, Oberkochen, Germany) was used for SEM imaging combined with EDS mapping.

### 2.7 Animal study

Five‐week‐old Balb/c or athymic nude mice (Raon Bio, Yongin, Republic of Korea) were used for in vivo experiments. All studies using animals conformed to the Guidelines for the Care and Use of Laboratory Animals of the Institute of Laboratory Animal Resources at Seoul National University (approved animal experimental protocol number, SNU‐190821‐5).

### 2.8 In vivo imaging

The biodistribution of macrocyclam derivatives was determined by MRI and positron emission tomography/computed tomography (PET/CT) scanning. Five‐week‐old female Balb/c nude mice were subcutaneously inoculated with 1 × 10^6^ SCC7 cells on the dorsal left side. When tumor volumes reached 150 mm^3^, samples were intravenously administered. For in vivo MRI, Gd@NrMC or Gd@ErMC was injected at a Gd dose of 0.7 mg/kg. The mice were monitored in vivo at different time points using an MRI scanner (Bruker‐Biospin) equipped with an animal‐imaging coil. For PET/CT imaging, ^64^Cu@NrMC or ^64^Cu@ErMC was injected at a ^64^Cu dose of approximately 240 μCi/mouse, and the mice were monitored in vivo at different time point using a PET/CT scanner (Inveon microPET/CT scanner, Siemens Medical Solutions, Malvern, PA, USA).

### 2.9 In vivo safety

The in vivo safety of macrocyclam derivatives was determined by histological and serological analyses. Five‐week‐old Balb/c mice were intravenously administered with at Gd@NrMC or Gd@ErMC at a Gd dose of 3.5 mg/kg. Briefly, NrMC (470 nmol) or ErMC (650 nmol) was mixed with gadolinium chloride in sodium acetate buffer (pH 5.0) and incubated for 2 h at 80°C. Unloaded Gd was removed using a PD‐SpinTrap™ G‐25 column. The samples including 70 μg of Gd were intravenously administered to mice. After 1 week, animals (*n* = 4 per group) were euthanized, and their organs (heart, lung, liver, spleen, and kidney) and sera were collected for histopathological and biochemical evaluation. Extracted organs were fixed in 10% formalin solution for 24 h and embedded in paraffin. For hematoxylin and eosin staining, each organ section was immersed in filtered Harris hematoxylin for 10 s and then in eosin for 30 s. The stained slides were visualized using a Vectra 3.0 Automated Quantitative Pathology Imaging System (PerkinElmer, Hopkinton, MA, USA), and the images were analyzed using InForm v2.4.11. software (PerkinElmer). The serum concentrations of alanine aminotransferase (ALT), aspartate aminotransferase (AST), aspartate aminotransferase (ALP), blood urea nitrogen (BUN), and total bilirubin (TBIL) were measured using an automatic chemistry analyzer (DRI‐CEHM 3500s; Fujifilm, Kanagawa, Japan).

### 2.10 Statistical analysis

Experimental data were compared by two‐sided, one‐way analysis of variance (ANOVA) using a post hoc Student–Newman–Keuls test. All statistical analyses were performed using SigmaStat software (version 12.0; Systat Software, Richmond, CA, USA). A *p* value <0.05 was considered statistically significant.

## 3 RESULTS

### 3.1 Characterization of metal‐loaded macrocyclam derivatives

The physicochemical properties of Gd‐complexed macrocyclam derivatives were characterized. Gd chelation on macrocyclam derivatives was confirmed by an arsenazo colorimetric method (Figure 2d). The gadolinium loading capacities of Gd@NrMC and Gd@ErMC were found to be 39.6 ± 17.9 and 29.3 ± 14.5 mol%, respectively. T1‐weighted MR imaging showed that the increase in contrast was concentration dependent (Figure 2e). The longevity relaxivities (*r*1) of Gd@NrMC and Gd@ErMC were calculated to be 41.2 and 33.4 mM^−1^ s^−1^, respectively (Figure 2f). The hydrodynamic sizes of Gd@NrMC (Figure 2g) and Gd@ErMC (Figure 2h) were 256.6 ± 41.9 and 310.2 ± 50.2 nm, respectively. Nanostructure of Gd@ErMC was maintained in physiological conditions (Figure S1). Measurements of their zeta potential showed that both Gd@NrMC and Gd@ErMC were slightly positive, suggesting a mild cationic charge at the particle surface (Figure 2i).

### 3.2 Cytotoxicity of metal‐loaded macrocyclam derivatives

**FIGURE 3:**
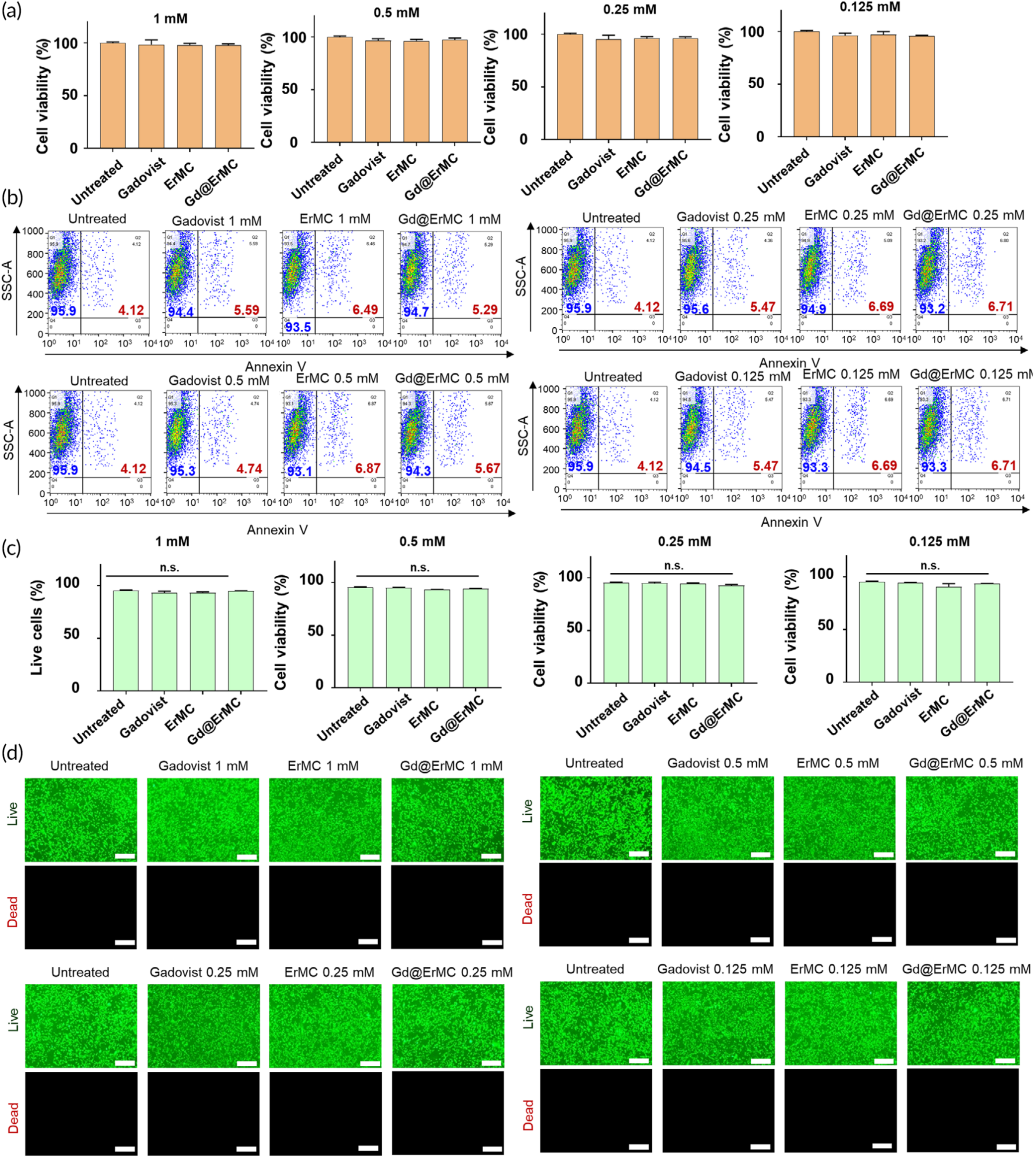
In vitro cytotoxicity of macrocyclam derivatives. (a) Viability of cells following treatment with various formulations. (b) Flow cytometry analyses of cells treated with macrocyclam derivatives and stained with Annexin V. (c) Quantitative analysis of plotting of Annexin V‐negative cell population. (d) Fluorescence microscopy assessment of live (green)/dead (red) 3T3 cells. Scale bar: 50 μm

**FIGURE 4:**
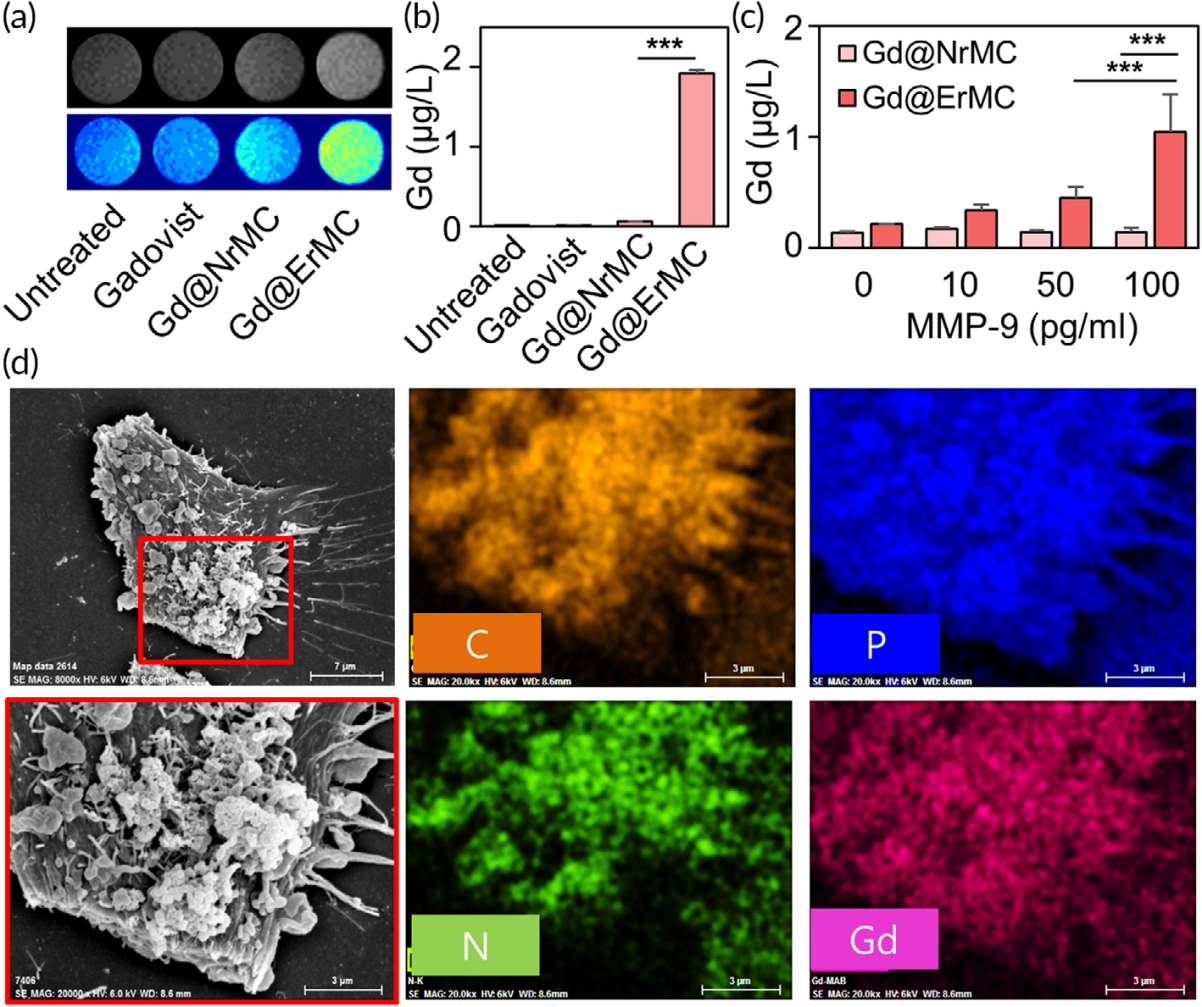
Cellular uptake of Gd delivered by macrocyclam derivatives. (a) T1‐weighted images of cells treated with various formulations of Gd. (b) Intracellular quantification of Gd determined by inductively coupled plasma mass spectrometry (ICP‐MS) after treatment with various formulations of Gd (***p < 0.001). (c) Intracellular quantification of Gd after treatment with various formulations in the presence of matrix metalloproteinases (MMP)‐spiked media (***p < 0.001). (d) Scanning electron microscopy combined with energy‐dispersive x‐ray spectroscopy (EDS‐SEM) of cells treated with Gd@ErMC. Elemental mapping was performed with carbon (C), phosphorus (P), nitrogen (N) and gadolinium (Gd).

The in vitro toxicity of macrocyclam derivatives was evaluated by various methods. MTT assays showed that the Gadovist and macrocyclam derivative‐based contrast agents had no effect on the viability of 3T3 cells (Figure 3a). Evaluation of the apoptosis of cells treated with macrocyclam derivatives by flow cytometry after staining with annexin V showed that none of these derivatives induced cell apoptosis (Figure 4b). Annexin V‐negative cell population occupied the majority, and there was no significant difference between groups (Figure 4c). These findings were confirmed by fluorescence live/dead cell staining, which showed that 3T3 cells retained full viability following treatment with macrocyclam derivatives (Figure 4d).

### 3.3 Enzyme‐responsive cellular uptake

Cellular uptake of Gd was evaluated by in vitro MRI, ICP‐MS, and EDS‐SEM imaging. T1 imaging of MMP‐overexpressing cells showed that MRI signals were higher for the Gd@ErMC group than for other groups (Figure 4a). Moreover, Gd concentrations were 32.2‐fold higher in Gd@ErMC‐treated than in Gd@NrMC‐treated cells (Figure 4b). Enzyme selective cellular uptake was confirmed by comparing Gd levels in cells grown in MMP‐spiked and blank media (Figure 4c). Evaluation of cells bearing Gd@ErMC showed that Gd accumulation was 7.2‐fold higher in cells treated with MMP than in blank media, whereas evaluation of cells bearing Gd@NrMC showed no significant difference in Gd uptake in the presence or absence of MMP. In addition, EDS‐SEM imaging of Gd@ErMC‐treated cells confirmed the colocalization of Gd on the cell surface, indicating that Gd@ErMC had bound to cell membranes (Figure 4d).

### 3.4 In vivo distribution of macrocyclam derivatives

**FIGURE 5:**
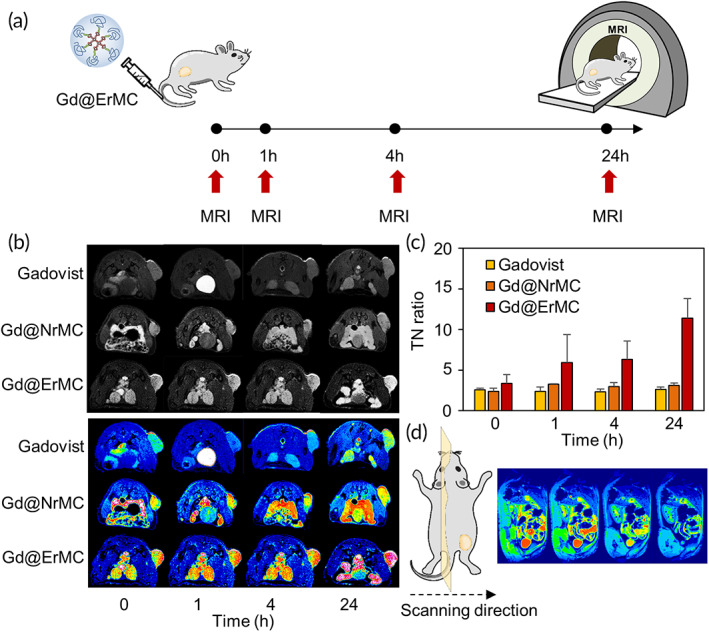
MR imaging of mice injected with macrocyclam derivatives. (a) Schematic diagram of sample treatment and imaging schedule. (b) T1‐weighted MR imaging of mice treated with Gadovist, Gd@NrMC and Gd@ErMC. (c) Tumor/normal tissue (TN) ratios of Gd, as determined by T1‐weighted signal intensity. (d) MR imaging of Gd@ErMC‐treated mice in the sagittal plane

The tumor‐specific deposition of Gd was assessed by T1‐weighted MRI following intravenous injection of Gd‐loaded macrocyclam derivatives into tumor bearing mice (Figure 5a). Cross‐sectional images showed that Gadovist treated mice had a strong signal at the bladder 1 h later, indicating rapid clearance of Gd through the kidneys (Figure 5b). In contrast, bladder signals were not clearly observed in mice treated with Gd@NrMC and Gd@ErMC. Signal intensity at tumor was enhanced 24 h after injection with Gd@ErMC. The tumor/normal tissue (TN) ratio in these mice increased from 1.8 at 1 h to 3.4 at 24 h, whereas that of mice treated with Gadovist and Gd@NrMC was not enhanced over time (Figure 5c). Sagittal views also indicated significant Gd accumulation in tumors (Figure 5d).

**FIGURE 6:**
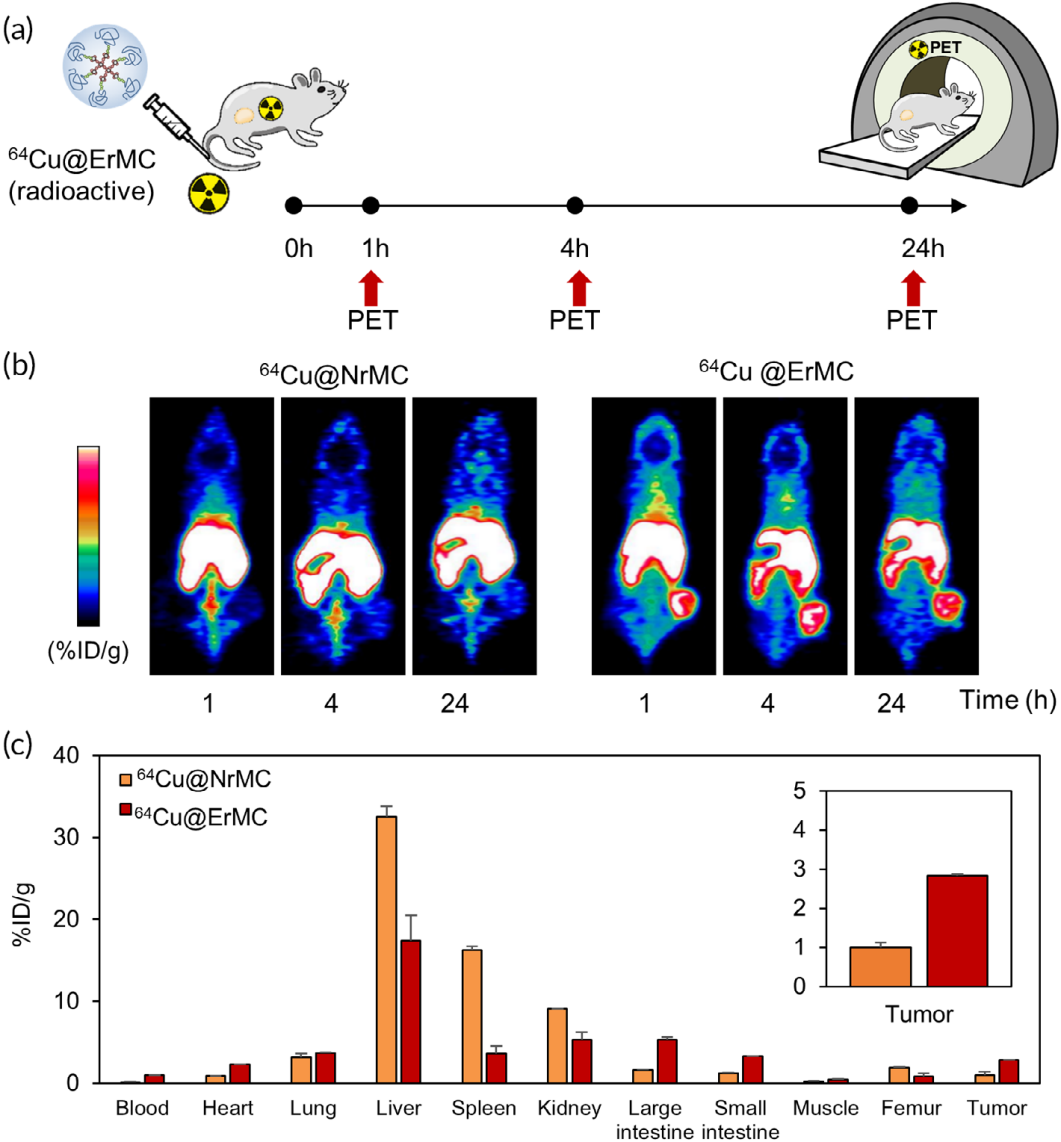
Positron emission tomography (PET) imaging of mice injected with macrocyclam derivatives. (a) Schematic diagram of sample treatment and imaging schedule. (b) PET images of mice treated with ^64^Cu@NrMC and ^64^Cu@ErMC. (c) The mean percent injected dose of ^64^Cu per gram in major organs of treated mice.

The ability of ErMC to enhance tumor detection on PET imaging was further evaluated by loading radioactive copper‐64 chloride into ErMC, yielding ^64^Cu@ErMC as a contrast agent (Figure 6a). The copper‐64 chloride loading capacities of ^64^Cu@NrMC and ^64^Cu@ErMC were found to be 20.5% and 22.4%, respectively. Copper chelation activity of macrocyclic complex was not affected by serum condition (Figure S2). The overall pictures showed enhanced signals in the liver and spleen, as well as in tumors, of mice treated with ^64^Cu@ErMC, whereas negligible signals were detected in tumor tissue of ^64^Cu@NrMC‐treated mice (Figure 6b). The percentage of injected ^64^Cu was 2.9‐ or 2‐fold higher in tumors of ^64^Cu@ErMC‐treated mice than of ^64^Cu@NrMC‐treated mice at 24 h or 48 h post‐dose, respectively, suggesting that ErMC improved contrast performance (Figure 6c and Figure S3).

### 3.5 In vivo toxicity of macrocyclam derivatives

**FIGURE 7:**
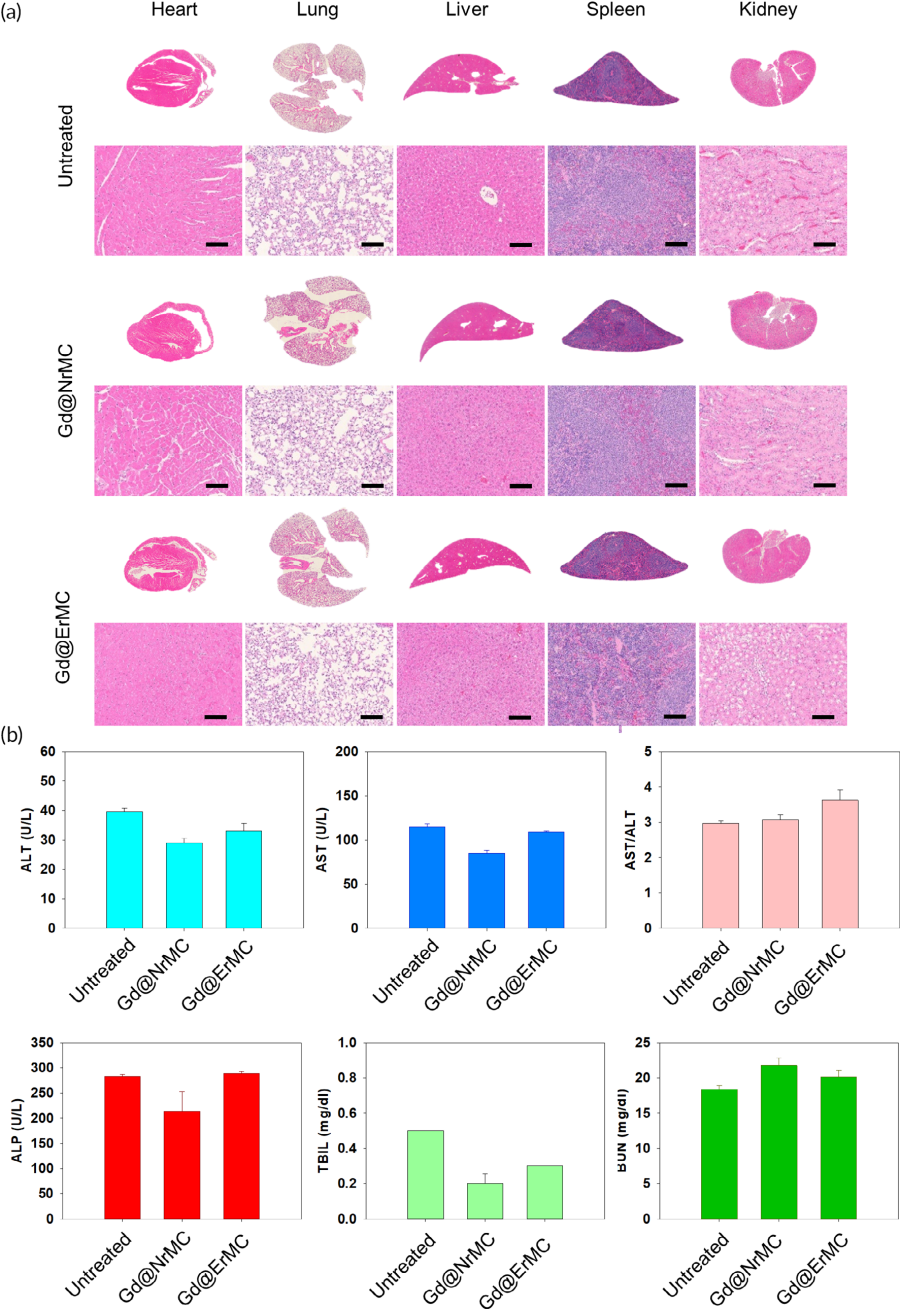
In vivo safety of macrocyclam derivatives. H&E‐stained sections of major organs (heart, lung, liver, spleen, and kidney). Scale bar: 100 μm. (a) Biochemical analysis of blood (b) extracted from macrocyclam‐treated mice

The systemic toxicity of macrocyclam derivatives was evaluated by examining the histology of major organs. At a Gd dose of 3.5 mg/kg, mice treated with Gd@NrMC or Gd@ErMC showed no visible microscopically abnormal features in the heart, lungs, liver, spleen and kidneys, although considerable accumulation of macrocyclam derivatives was observed in these organs (Figure 7a). Evaluation of liver and kidney functions in Gd@ErMC‐treated mice by measuring enzyme levels in blood 1 week after injection showed that enzyme levels were within normal range when compared with untreated mice (Figure 7b). These findings suggested that macrocyclam treatment did injure major organs or cause liver dysfunction.

## 4 DISCUSSION

The present study examined the capacity of a novel imaging probe based on macrocyclam derivatives to selectively deliver metal ions to solid tumors. Binding of a macrocyclam to an enzyme‐responsive moiety facilitated the self‐assembly of polymeric nanoparticles in an aqueous environment. Structurally, these nanoparticles were composed of condensed macrocyclam rings, probably due to the hydrophobic interactions and pi–pi stacking of the benzene rings. The shell layer was formed by a PEGylated peptide with outer exposure of the PEG chain. PEG promoted the physical stability of the nanoparticles and prevented protein opsonization while circulating in blood.^13^ PEG was therefore pivotal in extending the half‐life of nanoparticles by blocking RES‐mediated phagocytic clearance.

Gd@NrMC and Gd@ErMC theoretically bear the same Gd‐loading capacity due to sharing the same structure of macrocyclam core. However, ErMC is equipped with tumor enzyme‐cleavable peptides, which increase rigidity of whole molecule and interferes into the coordination of Gd and macrocyclam at certain level. Indeed, previous study has indicated that increase in the rigidity of chelator may result in slower complex formation kinetic.^14^ Therefore, ErMC may posed a lower Gd‐loading capacity compared to nonpeptide modified NrMC in a same synthesis condition. In this study, we chose Gd@ErMC by not only the Gd‐loading capacity but also the ultimate aim of tumor‐specific accumulation via enzyme‐cleavable peptide. The lower Gd‐loading capacity could be compensated by the adjustment in administered dose. By both in vitro and in vivo model, we have proved that treatment at the same dose of Gd, Gd@ErMC showed the higher cellular uptake as well as tumor accumulation.

MMP is a factor in the TME that has been targeted in designs of drug delivery systems.^15^, ^16^ TME‐sensitive platforms have been formulated by engineering nanoparticles, such as liposomes, graphene nanosheets, and gold nanoparticles, with MMP‐responsive peptides to improve tumor distribution.^10^, ^17^, ^18^ In the present study, the introduction of an MMP‐responsive peptide between each PEG chain and the macrocyclam provided a trigger for deliberate shedding of the PEG layer in the presence of MMPs. The interaction of MMPs and their peptide substrates tethered to a macrocyclam increased the time the contrast agent remained at the tumor site.

Enzyme‐responsive cleavage of ErMC resulted in the exposure of the positively charged macrocyclic cyclam and induced charge interactions between the tumor cell membrane and particle. A bicyclam derivative (plerixafor) has been approved for hematopoietic stem cell mobilization in the treatment of non‐Hodgkin's lymphoma.^19^ By forming complexes with Zn^2+^, plerixafor inhibits CXCR4 and prevents peripheral stem cells from homing to the bone marrow.^20^ Plerixafor derivatives have been found to increase the tumor homing effect of modified nanoparticles by targeting CXCR4 expressed on tumor cells.^21^ Eight cyclams of ErMC showed high cellular uptake of metal ions in an enzyme‐rich environment.

Relaxivity is one of the key parameters by which the contrast performance of GBCAs can be evaluated by MRI.^22^, ^23^ Incorporation of Gd into a polymer, protein or nanoparticle has been found to significantly increase their contrast performance.^24^, ^25^, ^26^, ^27^ Several mechanisms may explain the enhanced relaxivity when immobilizing Gd in the macrostructures. First, compared with a single chelated‐Gd complex, the number of Gd atoms per nanoparticle would be much higher. That would increase the relaxivity density per particle, assuming one unit of delivery successfully entered tumor tissue. Second, the relaxivity of Gd was also dependent on the global rotation motion of the chelator.^28^ Tethering the structure of chelators to a polymer or incorporating them into nanoparticles has been reported to dramatically promote *r*1 relaxivity because the macromolecular structure limited the rotational motion of Gd.^29^ In the present study, Gd ions were chelated with macrocyclam in the core of nanoparticles, which limited the global rotational motion, thus increasing their relaxivity. By incorporating Gd into ErMC, the optimal dose of Gd would be lower, thereby reducing its toxicity.

Currently approved Gd contrast agents primarily based on the complexation of gadolinium and small molecule structure, for example, Gadovist®, Primovist®, Magnevist®, and so on. There is still limitation in their tumor diagnosis efficacy because of fast clearance, poor tumor retention, and nonspecific accumulation at normal tissue. The design of tumor enzyme‐responsive macrocyclam is to provide a tumor‐targeted platform for intravenous contrast agent delivery. There are three key factors in platform design of ErMC to enhance their ability as a contrast agent. Firstly, the structure of macrocyclam provided multiple pockets to carry Gd ions in a single molecular complex. This feature allows more Gd delivered to tumor cells when ErMC complex is taken up. Second, by polymerizing the cyclam rings in to macrocyclam, the whole complex has molecular size increased and thus being supposed to have less rotational motion. This feature is important to improve the overall relaxation efficiency of Gd‐based contrast agents.^29^ Third, PEG modification provides a stealthy effect for nanoparticle and protects them from nonspecific uptake. ErMC was protected with PEGylated peptide, thus being geared with the stealth effect when they are traveling in blood stream. Of note, once the ErMC particles extravasate and accumulate at tumor tissue, the shedding of PEG shield via cutting off the peptide chain is mediated by tumor enzymes. The unshielded macrocyclam core was then internalize into tumor cells and extend their retention in tumor tissue. Collectively, these features are combined to in single platform to yield Gd@ErMC with enhanced contrast performance as compared with current Gd‐based imaging agents.

The macrocyclam derivative structure contains six cyclam rings, creating multiple sites for the chelation of metal ions such as Gd^3+^ and ^64^Cu^2+^. Due to the high chelation affinity of the eight cyclam core in ErMC, Gd@ErMC was stable and nontoxic, both in vitro and in vivo. The high affinity of the cyclam ring to these metal ions would prevent the release of free ions from the nanoparticles while traveling through tissue.

**FIGURE 8:**
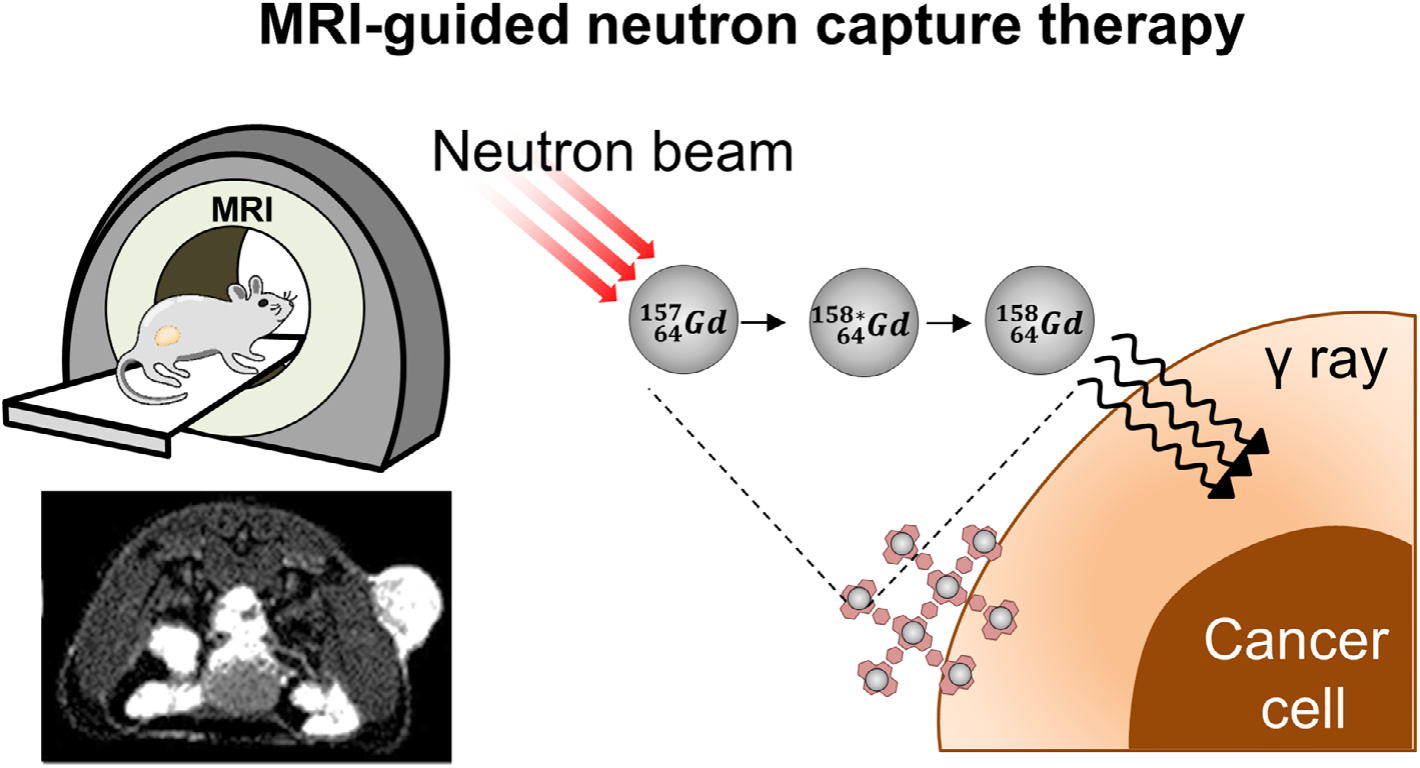
Proposed strategy for the use of metal‐macrocyclam complexes for image‐guided radiotherapy

Moreover, Gd‐based neutron capture therapy (GNCT) requires tumor accumulation and retention of Gd.^30^ Unlike conventional Gd‐based contrast agents, which lack targeting ability and are expelled rapidly, ErMC‐based contrast agent might accumulate in enzyme‐rich target tissues, allowing image‐guided radiotherapy (Figure 8).

## 5 CONCLUSION

In the present study, a novel platform was developed as a carrier for metal ions bound to macrocyclam rings that facilitate imaging performance in MRI and PET. The strategy of inserting an enzyme‐responsive peptide between the PEG chain and macrocyclam derivative to create an enzyme‐rich environment responsive system induced a more specific distribution of contrast agent in target tissue. This platform has the potential to provide an effective way to image a target lesion more precisely and opens up further applications of ErMC as a theranostic agent for MR‐guided radiotherapy. Although our study has only tested the delivery performance of ErMC using Gd and ^64^Cu as contrast agents for MRI and PET, its application could also be extended to other imaging metallic radionuclides that have potential chelating affinity with cyclam ring such as gallium, technetium, zirconium, and yttrium. On the other side, further dose selection of these materials depending on tumor type, tumor enzyme expression level should be evaluated. Also, the ability of small tumor tracking or tumor metastasis needs to be accessed to optimize the applications in solid tumor diagnosis.

## AUTHOR CONTRIBUTIONS

**Quoc‐Viet Le:** Methodology (equal); visualization (equal); writing – original draft (equal). **Jaiwoo Lee:** Data curation (equal); formal analysis (equal); methodology (equal); visualization (equal). **Seungbeom Ko:** Conceptualization (supporting); data curation (equal); software (lead); visualization (supporting). **Hyunjung Kim:** Data curation (equal); methodology (equal). **Thien Y Vu:** Software (supporting); visualization (supporting). **Yearn Seong Choe:** Methodology (equal); resources (equal); supervision (supporting). **Gayong Shim:** Conceptualization (equal); funding acquisition (lead); supervision (equal); writing – review and editing (equal). **Yu‐Kyoung Oh:** Funding acquisition (equal); supervision (equal); writing – review and editing (equal).

## CONFLICT OF INTEREST

The authors declare no potential conflicts of interest.

## Supporting information


**Data S1:** Supporting Information

## Data Availability

Data available on request from the authors.
