# Supplementary material for: Enzyme‐responsive macrocyclic metal complexes for biomedical imaging
Source: Bioeng Transl Med. 2022 Dec 21;8(5):e10478. doi: 10.1002/btm2.10478 (PMC10487310; doi:10.1002/btm2.10478)
Supplement: Supplementary file 1 — Data S1: Supporting Information [file BTM2-8-e10478-s001.docx]

***Supporting Information***

***Enzyme responsive macrocyclic metal complexes for biomedical imaging***

Quoc-Viet Le^†,1^, Jaiwoo Lee^†,2^, Seungbeom Ko^†,2^, Hyunjung Kim^3^, Thien Y Vu^1^, Yearn Seong Choe^3,4^, Yu-Kyoung Oh^2,*^, Gayong Shim^5,*^

^1^Faculty of Pharmacy, Ton Duc Thang University, Ho Chi Minh City, Vietnam

^2^College of Pharmacy and Research Institute of Pharmaceutical Sciences, Seoul National University, 1 Gwanak-ro, Gwanak-gu, Seoul 08826, Republic of Korea

^3^Department of Nuclear Medicine, Samsung Medical Center, Sungkyunkwan University School of Medicine, Seoul, Republic of Korea

^4^Department of Health Sciences and Technology, SAIHST, Sungkyunkwan University, Seoul, Republic of Korea

^5^School of Systems Biomedical Science and Integrative Institute of Basic Sciences, Soongsil University, Seoul 06978, Republic of Korea

†The authors equally contributed.

* Corresponding author (Tel: 82-2-820-0451)

E-mail addresses: ohyk@snu.ac.kr (Y.K. Oh) and shim@ssu.ac.kr (G. Shim)

***
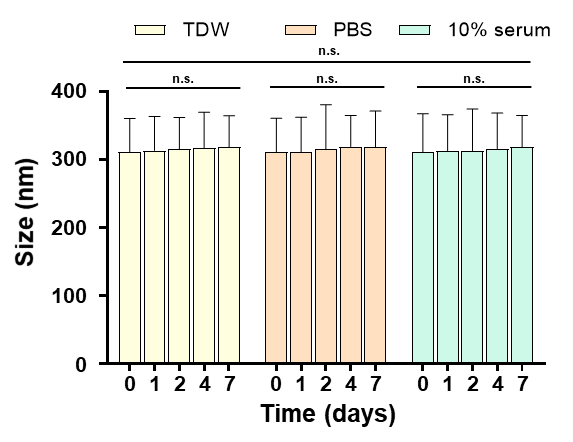
***

**Fig. S1. Serum stability of macrocyclic complex**

Size of ErMC was measured in TDW, PBS, or 10% serum condition.

***
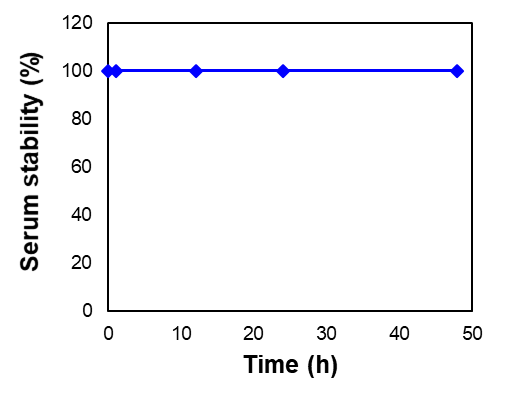
***

**Fig. S2. Serum stability of ^64^Cu in macrocyclic complex**

Stability of ^64^Cu in ErMC was observed in 50% serum condition.

***
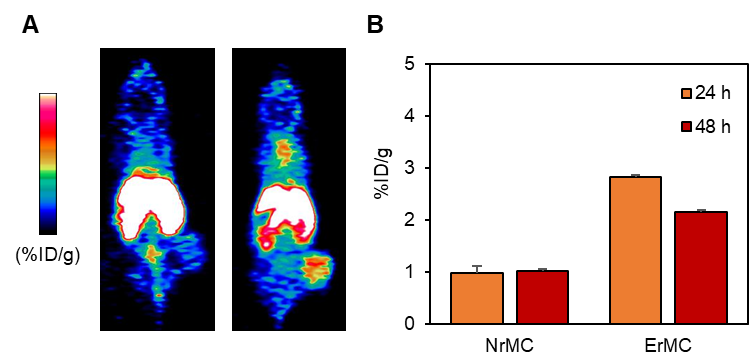
***

**Fig. S3. PET imaging of ^64^Cu in macrocyclic complex**

(A) PET images of mice treated with 64Cu@NrMC and 64Cu@ErMC were obtained at 48 h post-dose. (B) The mean percent injected dose of ^64^Cu per gram in tumor tissue of treated mice.
